# Mechanistic insights into mode of action of novel natural cathepsin L inhibitors

**DOI:** 10.1186/1471-2164-14-S8-S10

**Published:** 2013-12-09

**Authors:** Chetna Tyagi, Sonam Grover, Jaspreet Kaur Dhanjal, Sukriti Goyal, Manisha Goyal, Abhinav Grover

**Affiliations:** 1 Apaji Institute of Mathematics & Applied Computer Technology, Banasthali University, Tonk, Rajasthan, India - 304022; 2 School of Biotechnology, Jawaharlal Nehru University, New Delhi, India - 110067; 3 Department of Biotechnology, Delhi Technological University, New Delhi, India - 110042

**Keywords:** Cancer, 3D QSAR, pharmacophore, thiosemicarbazone, cathepsin L, inhibitor

## Abstract

**Background:**

Development of a cancerous cell takes place when it ceases to respond to growth-inhibiting signals and multiplies uncontrollably and can detach and move to other parts of the body; the process called as metastasis. A particular set of cysteine proteases are very active during cancer metastasis, Cathepsins being one of them. They are involved in tumor growth and malignancy and have also been reported to be overexpressed in tumor cell lines. In the present study, a combinatorial approach comprising three-dimensional quantitative structure-activity relationship (3D QSAR), ligand-based pharmacophore modelling and search followed by cathepsin L structure-based high throughput screening was carried out using an initial set of 28 congeneric thiosemicarbazone derivatives as cathepsin L inhibitors. A 3D QSAR was derived using the alignment of a common thiosemicarbazone substructure. Essential structural features responsible for biological activity were taken into account for development of a pharmacophore model based on 29 congeneric thiosemicarbazone derivatives. This model was used to carry out an exhaustive search on a large dataset of natural compounds. A further cathepsin L structure-based screen identified two top scoring compounds as potent anti-cancer leads.

**Results:**

The generated 3D QSAR model showed statistically significant results with an r^2 ^value of 0.8267, cross-validated correlation coefficient q^2 ^of 0.7232, and a pred_r^2 ^(r^2 ^value for test set) of 0.7460. Apart from these, a high F test value of 30.2078 suggested low probability of the model's failure. The pharmacophoric hypothesis chosen for searching the natural compound libraries was identified as DDHRR, where two Ds denote 2 hydrogen donors, H represents a hydrophobic group and two Rs represent aromatic rings, all of which are essential for the biological activity. We report two potential drug leads ZINC08764437 (NFP) and ZINC03846634 (APQ) obtained after a combined approach of pharmacophore-based search and structure-based virtual screen. These two compounds displayed extra precision docking scores of -7.972908 and -7.575686 respectively suggesting considerable binding affinity for cathepsin L. High activity values of 5.72 and 5.75 predicted using the 3D QSAR model further substantiated the inhibitory potential of these identified leads.

**Conclusion:**

The present study attempts to correlate the structural features of thiosemicarbazone group with their biological activity by development of a robust 3D QSAR model. Being statistically valid, this model provides near accurate values of the activities predicted for the congeneric set on which it is based. These predicted activities are good for the test set compounds making it indeed a statistically sound 3D QSAR model. The identified pharmacophore model DDHRR.8 comprised of all the essential features required to interact with the catalytic triad of cathepsin L. A search for natural compounds based on this pharmacophore followed by docking studies further screened out two top scoring candidates: NFP and AFQ. The high binding affinity and presence of essential structural features in these two compounds make them ideal for consideration as natural anti-tumoral agents. Activity prediction using 3D QSAR model further validated their potential as worthy drug candidates against cathepsin L for treatment of cancer.

## Background

Cancer is a condition characterized by unregulated growth and division of cells that have become abnormal and can invade adjoining parts of the body. Cancerous cells arise as a consequence of mutations in the critical genes. According to the world cancer report, an estimated number of 7.6 million fatalities were recorded in 2008 and 12.7 million new cases were diagnosed. This number is expected to rise to 21 million by 2030 [1]. A series of proteolytic enzymes are a pre-requisite for the tumor cells to undergo metastasis in which tumor cells travel to distant organs and form new tumors [2-6]. Cysteine proteases are a group of such proteolytic enzymes that are characterized by a cysteine residue in their active site region [7-13]. Cathepsins are a subfamily of 11 human lysosomal cysteine proteases included in the papain family [14]. Most of them have been found to be involved in tumor growth and malignancy. Cathepsin L is a globular endopeptidase which plays an important role in vital physiological processes and is reported to be overexpressed in various human tumors [15-17]. Knowledge of this family of proteases and their inhibitors can prove to be a major breakthrough in cancer management and thus is the subject of interest for the present study [18]. Various inhibitors have been characterized and studied extensively against cathepsins, for e.g. nitriles [19], azepanone analogues [20] and disulfides [21] among others. In the present study we focus on the thiosemicarbazone moiety that has been utilized previously in the development of anticancer agents by inhibition of cathepsin L.

Thiosemicarbazones incorporate an important class of N, S-donor ligands [22], and are basically schiff bases obtained by condensation of thiosemicarbazides with an aldehyde or ketone [23]. They first appeared in the 50's as drugs against tuberculosis and leprosy [24,25]. Later, their antiviral properties were reported which led to a huge research in this area resulting in commercialization of methisazone also named as Marboran, to treat smallpox [26]. Benzophenone thiosemicarbazone derivatives have earlier been reported as potential therapeutics against malaria, sleeping sickness and chagas' disease [27-30]. Recently, antitumor activity of KGP94, a functionalized benzophenone thiosemicarbazone derivative, was evaluated for breast cancer against cathepsin L [31]. Triapine (3-aminopyridine-2-carboxaldehyde thiosemicarbazone) has already been evaluated as ribonucleotide reductase inhibitor for anticancer therapy [32]. Apart from these, various other derivatives of thiosemicarbazones such as thiophene, pyridine and fluorene have also been tested as inhibitors of cathepsin L and their IC_50 _values have been reported [33,34].

A fast and accurate approach to search for novel therapeutics against various cancers is the need of the hour. *In silico *methods involving ligand based drug design are viable approaches to speed up the drug discovery process. 3D QSAR has emerged as a robust technique in rational drug design to predict the biological activities of the prospective inhibitors using the knowledge of three-dimensional properties of the ligands through a chemometric approach. It develops statistically significant models to guide synthesis of novel inhibitors on the assumption that the extent of receptor binding directly relates to its biological activity [35,36]. In 3D QSAR, molecular structures are represented by a set of numbers called as descriptors. For QSAR model development, the receptor binding site is considered to be rigid and the ligand molecules should belong to a congeneric series [37]. From a pool of molecular descriptors, optimal variables are chosen using a stochastic method. Molecular fields, which are basically steric and electrostatic interaction energies, are calculated and a molecular field analysis model is predicted [38]. The model thus generated is evaluated for its robustness by determining its capacity to predict the activity of compounds not belonging to the training set. This validation is done based on the calculation of statistical parameters. On the other hand, a pharmacophore is a molecular framework that carries the essential features responsible for a drug's biological response [39]. Features like aromatic rings, hydrogen donors and acceptors, hydrophobes and positively and negatively ionisable chemical groups are marked and the resulting pharmacophoric hypothesis is scored for its validity. Natural compounds in good alignment with such a hypothesis can be taken as potent drug leads.

In this study, a congeneric dataset comprising of 28 thiosemicarbazone derivatives was first chosen to build a 3D QSAR model that evaluates the activity of the ligands against cathepsin L. And we also find out the molecular features essential for their activity using the pharmacophore model. Despite the continuous efforts in the direction of finding novel cathepsin L inhibitors, there are no clinical agents available in human clinical trials yet [31]. This study establishes the use of thiosemicarbazone derivatives by contributing towards understanding its essential characteristics as potent anti-cancer candidate and thus paves way for an accelerated evaluation of novel thiosemicarbazone-based lead candidates using the predicted QSAR model.

## Materials and methods

### Compound dataset for model development

In this study, a congeneric series of thiosemicarbazone derivatives with inhibitory properties against human cathepsin L were selected for 3D-QSAR model development [33,34]. The 2D structures of the template molecule and 61 derivatives were drawn using Chemsketch [40] which were then aligned with the most active molecule (reference molecule). A total of 28 molecules were selected on alignment with the thiosemicarbazone template based on lower RMSD values, which indicate optimal alignment. These 2D structures were converted to 3D using Vlife Engine platform of VLifeMDS [41] and later energy minimized using the force field batch minimization utility with default parameters. These optimized compounds were finally used for 3D-QSAR model development.

### Computation of force field

The 28 aligned compounds along with their pIC50 values were given as input for force field calculation. For 3D QSAR, a force field was computed keeping default grid dimensions and including steric, electrostatic and hydrophobic descriptors while keeping dielectric constant at the default value (1.0). The charge type chosen for computation was Gasteiger-Marsili. The values calculated for the descriptors along with their grid points were arrayed upon the worksheet and the invariable columns were removed using QSAR tools.

### Model development

Using advanced data selection wizard, the column containing the activity values (pIC50) of the compounds was selected as the dependent variable and the rest as independent variables. After manual selection of the test set, the unicolumn statistics of both the test and the training sets were calculated. This analysis provided validation of the chosen training and test sets. A critical step in QSAR model development is the selection of optimal variables from the available set of descriptors which set out a statistically significant correlation of the structure of compounds with their biological activity. Using the variable selection and model building wizard, the model was built by stepwise-forward method [42]. All the values were kept default except the number of descriptors in the final equation which was changed to 4 and variance cut-off as 0.1. This variable selection method can be combined with a number of different regression analysis techniques like partial least squares (PLS) [43], partial component regression [44], k nearest neighbour [45] among others by selecting the appropriate combination. In the present study, we report a 3D QSAR model built using PLS.

### Model validation

Many statistical parameters like n (number of compounds in regression), k (number of variables), degree of freedom, optimum component (number of optimum PLS components in the model), r^2 ^(squared correlation coefficient), F-test (Fischer's value), q^2 ^(cross-validated correlation coefficient), pred_r^2 ^(r^2 ^for external test set), Z score (randomisation test), best_ran_q^2 ^(highest q^2 ^value in the randomisation test) and best_ran_r^2 ^(highest r^2 ^value in the randomisation test) need to be taken into account to consider the model as a robust one. For a model to be statistically significant, the following conditions should be considered: r^2^, q^2 ^> 0.6 and pred_r^2 ^> 0.5 [1,2]. Since, F-test gives an idea of the chances of failure of the model, a value greater than 30 is considered to be good. On the other hand, low standard error values establish absolute quality of the model.

### Internal and external validation

For internal validation using leave-one-out method [46], the cross-validated coefficient, q^2 ^is calculated using the given equation:

$$q^{2}=1-\frac{\Sigma {\left(y_{i}-{ŷ}_{i}\right)}^{2}}{\Sigma {\left(y_{i}-y_{mean}\right)}^{2}}$$

where $y_{i}$ and ${ŷ}_{i}$ are the actual and predicted activities of the  ith molecule (i = 1-24 except 9: refer Additional file 1) in the training set, respectively, and $y_{mean}$ is the average activity of all the molecules in the training set.

For external validation, the pred_r^2 ^value that gives an account of the statistical correlation between predicted and actual activities of the test set compounds was calculated as follows:

$$\text{pred}\text{\_}r^{2}=1-\frac{\Sigma {\left(y_{i}-{ŷ}_{i}\right)}^{2}}{\Sigma {\left(y_{i}-y_{mean}\right)}^{2}}$$

where $y_{i}$ and ${ŷ}_{i}$ are the actual and predicted activities of the  ith molecule (i = 25-29: refer Additional file 1) in the test set, respectively, and $y_{mean}$ is the average activity of all the molecules in the training set.

To avoid the risk of chance correlation, Y randomisation test was carried out by comparing the resultant linear model with those derived from random data sets. Various models were built on random datasets generated by rearranging the molecules in the training set so as to compare them with the obtained 3D QSAR model on the basis of Z-score [47]. A Z-score value is calculated by the following formula:

$$\text{Z}\;\text{score}=\frac{\left(h-\mu \right)}{\sigma }$$

where  is the q^2 ^value calculated for the actual data set,  μ is the average q^2 ^and  σ is the standard deviation calculated for various models built on different random data sets.

### Pharmacophore-based virtual screening

Using the same set of compounds as taken for the 3D QSAR model development, we embarked upon a search for similar anti-cancer natural compounds. The essential features responsible for a molecule's biological activity are represented through a pharmacophoric hypothesis, which is then used for a rigorous search for compounds constituting the same features. The pharmacophore model was created using the Phase module of Schrodinger [48]. It is a 5-step procedure which is carried out by selecting the 3D optimized molecules, prepared using Ligprep and manually entering their activity values (pIC50). A number of hypotheses were generated along with their respective set of aligned conformations. Using Phase, an exhaustive search was done for a lead molecule based on the pharmacophore after selecting the best hypothesis amongst them.

### Virtual screening targeted against cathepsin L

The compounds screened after pharmacophore-based search were further evaluated for their inhibitory potency against Cathepsin L by using Schrodinger's Glide docking platform [49,50]. It works by creating a cubic grid (10 $\dot{\text{A}}$ side) around the user-specified critical residues and directing the approaching ligand at the specific site. An extra precision (XP) docking was carried out to screen 7409 compounds obtained after pharmacophore based screening, of which those lying above the specified threshold were chosen. XP docking serves the purpose of correlating good poses with good scores and discarding the false positives.

## Results and discussion

### 3D QSAR model

A 3D-QSAR model development works to find a statistical correlation between the structures and activity of chemical compounds by calculating 3D molecular descriptors which include steric, electrostatic and hydrophobic points marked on the 3D spatial grid. After selecting the Gasteiger-Marsili charges for computing the force field grid, the invariable columns were removed which reduced the descriptor number from 2971 to 2944. pIC50 was selected as the dependent variable while the calculated 3D descriptors were chosen as independent variables. The test set constituting the compounds A3, A5, A9, A19 and A34 (Additional file 1) was selected manually after which the unicolumn statistics were calculated for both the training and test set compounds.

### Unicolumn statistics

The unicolumn statistics analysis showed that the training and test sets were suitable for 3D-QSAR model development. For an appropriate model, max of the training set should be more than max of the test set and min of the test set should be higher than min of the training set. The unicolumn statistics scores are shown in Table 1. The max and min of the training and test sets were found in concurrence with the ranges specified and suggested that the test set was interpolative. Besides, the relative difference between the mean and point density distribution of the two sets was determined by the mean and standard deviation. In this case, the mean of the test set was a bit lower than the train set implying the presence of relatively moderate number of active molecules as compared to the inactive ones. Also, the similarity in standard deviation indicated that the spread with their respective means in both the sets were comparable.

**Table 1:** Unicolumn statistics for training and test set for Cathepsin L inhibiting compounds

Set	Column name	Average	Max	Min	Std Dev	Sum
Training	pIC50	6.5100	7.6400	5.310	0.7130	14.7297
Test	pIC50	6.3022	7.0990	5.580	0.6795	3.5110

### Linear model equation and validation with statistical parameters

Selecting the stepwise forward (SW) variable selection method, we built a 3D-QSAR model for which the details are given. The selected descriptors were E_86, E_943, E_463, and S_482, which represent steric and electrostatic field energy of interactions at their respective spatial grid points. No hydrophobic descriptor was found contributing in the final model obtained by the SW algorithm. The numbers in the chosen descriptors represented their positions on the 3D spatial grid. Equation 1 represents the obtained 3D QSAR model:

(1) $$\text{pI}{\text{C}}_{\text{5}0}=\text{3}.\text{89857}\left(\text{E}\text{\_}\text{86}\right)+\text{3}.\text{12363}\left(\text{E}\text{\_}\text{943}\right)-0.\text{114297}\left(\text{E}\text{\_}\text{463}\right)+0.0\text{1525}0\text{2}\left(\text{S}\text{\_}\text{482}\right)+\text{5}.\text{73211}$$

While each descriptor is accompanied by a numerical coefficient, the last single numerical value is the regression coefficient. This model was both internally and externally validated using the LOO method by calculating statistical parameters which are critical requirements for a model to be robust. The number of compounds in the training set was specified by N which is 23 in this case. Considering the correlation coefficient, r^2 ^(0.8267), cross-validated correlation coefficient q^2 ^(0.7232), pred_r^2 ^(0.7460), low standard error value, r^2^_se (0.3194), q^2^_se (0.4036) and pred_r^2^_se (0.3619), the model can be stated to be a robust one. Along with this, the F-test value (30.2078) implied that the model is 99 % statistically valid with 1 in 10000 chance of failure. Other important statistical parameters are presented in Table 2. Z-scores for r2, q2 and pred_r2 have been specified to emphasize its importance in QSAR model validation. Zscore_r2 of 5.55599 implies a 100% area under the normal curve. Zscore_q2 of 3.71813 implies a 99.99% area under the normal curve and Zscore_pred_r2 of 1.45442 implies a 92.70% area under the normal curve all of them indicating that the respective scores are not far away from the mean 'μ' and thus validate the model's statistical robustness.

**Table 2:** The statistical parameters calculated for developed 3D-QSAR model

Dep Variable	ZScore r2	ZScore q2	Best Rand r2	Best Rand q2	Alpha Rand r2	Alpha Rand q2	Z Score Pred r2	best Rand Pred r2	alpha Rand Pred r2
pIC50	5.55599	3.71813	0.56529	0.32339	0.0000	0.00100	1.45442	0.90479	0.100000

**Figure 1:**
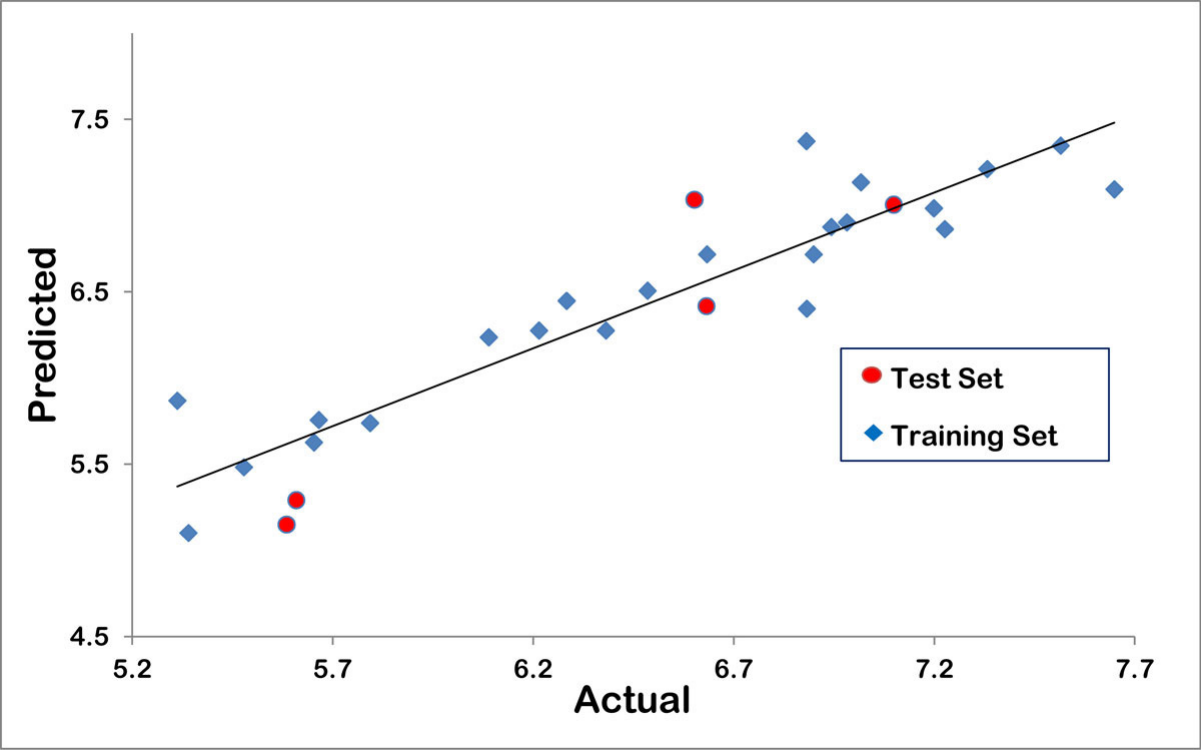
**Graphical representation of observed vs. predicted activity for training and test set**.

**Figure 2:**
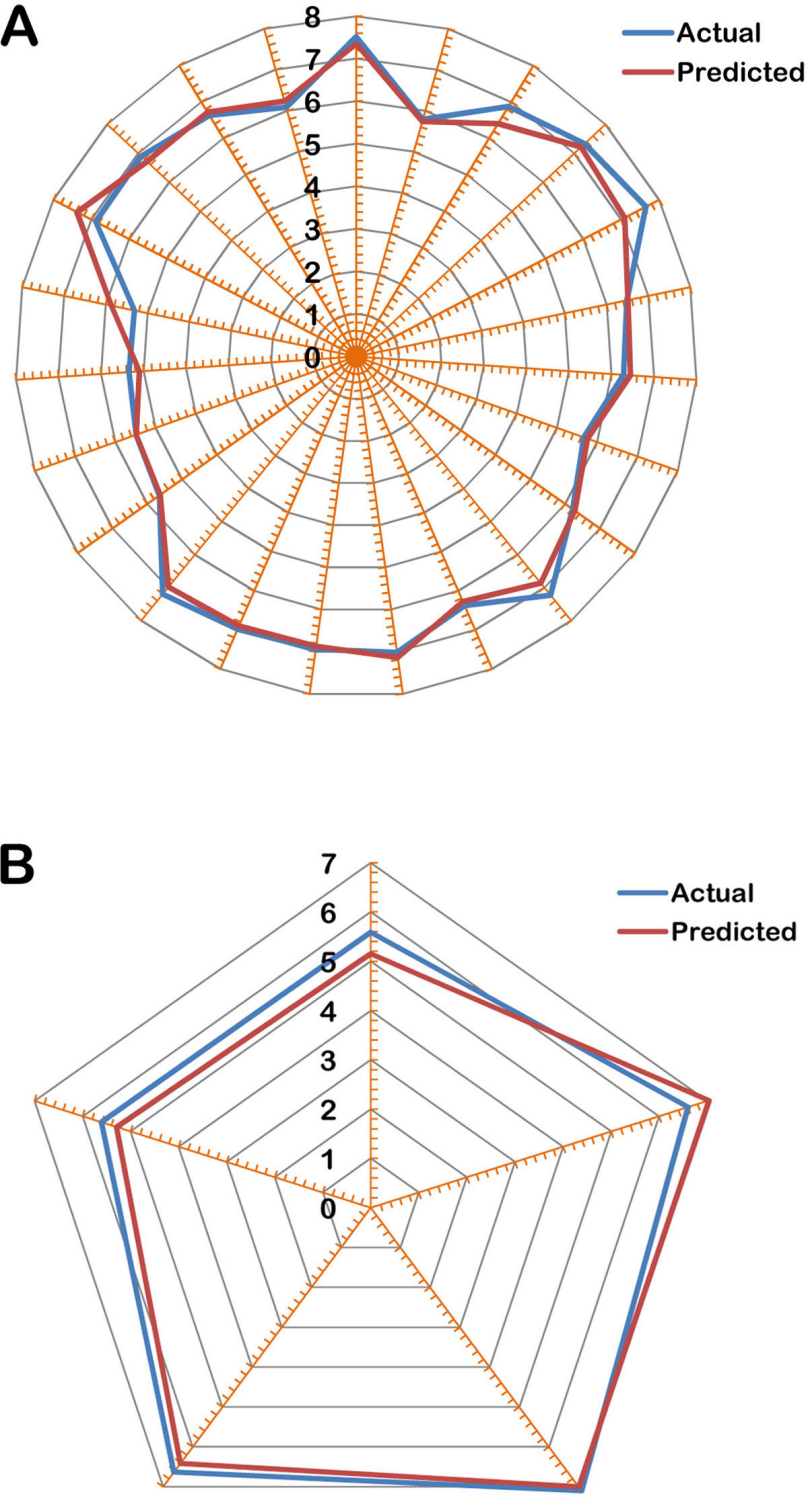
**Radar plots showing the observed and predicted activities for (a) training set (b) test set**.

**Figure 3:**
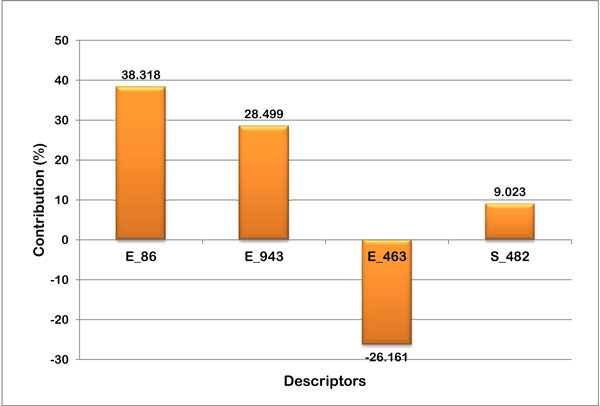
**Contribution plot of 3D descriptors of the generated QSAR model**.

The robustness of the model is better understood through the linear graphical representation between actual and predicted activities of the final 28 compounds (Figure 1) and radar plots for training and test sets (Figure 2 (a, b)). The linear graphical representation shows the extent of variation between the actual and predicted activities of the congeneric set. The larger the distance of training and test set points from the regression line, more is the difference between the actual and the predicted activity values. The radar graphs depict the difference in the actual and predicted activities for the training and the test sets separately by the extent of overlap between blue (actual activity) and red (predicted activity) lines. The radar plot for training set represents a good r^2 ^value if the two lines show a good overlap while for the test set a good overlap represents high pred_r^2 ^value. The contribution plot for each descriptor is given in Figure 3. The contribution of each descriptor specifies the properties that should be present in the drug lead for its enhanced inhibitory activity. Presence of descriptors with positive contribution increases its inhibitory activity while descriptors with negative contribution decrease the same. For electrostatic descriptors, a positive contribution indicates the requirement of electropositive group at that site and an electronegative group for negatively contributing descriptor.

**Figure 4:**
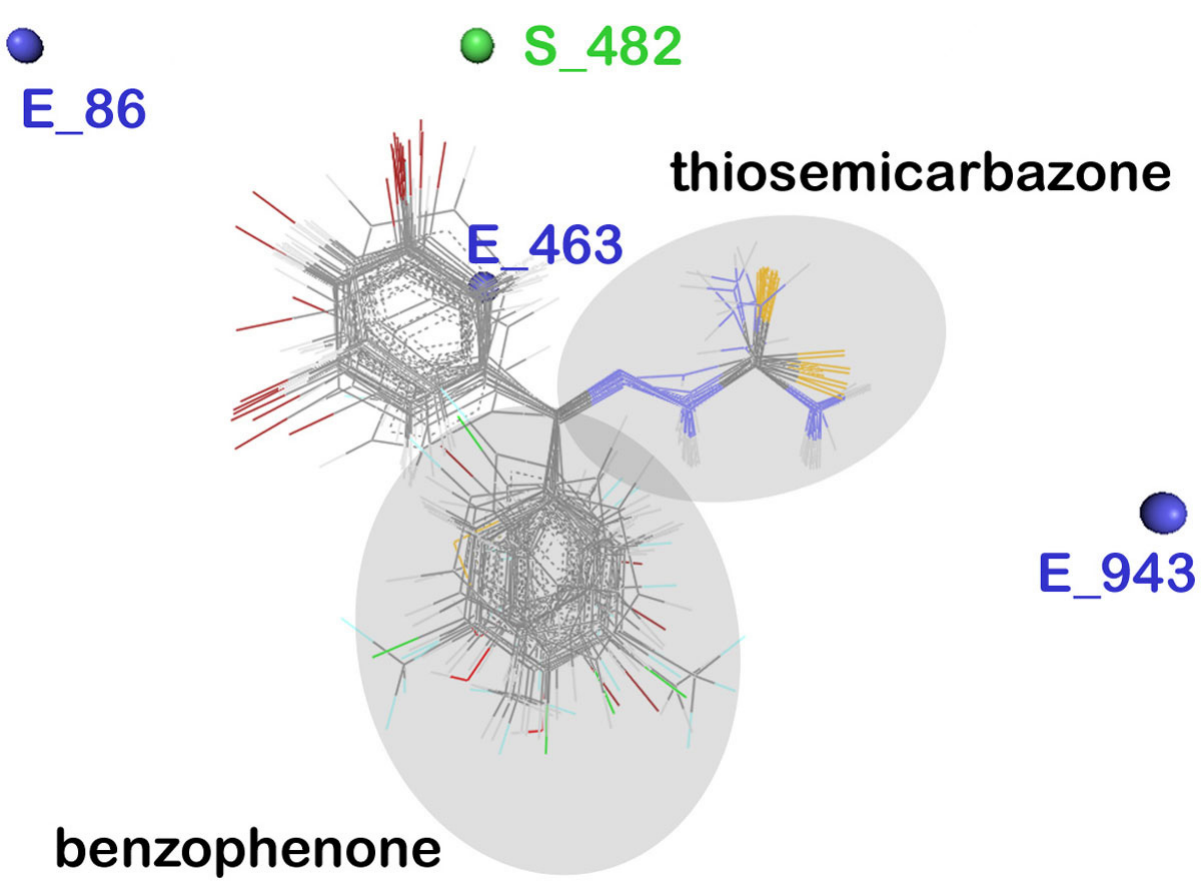
**Depiction of aligned congeneric set of molecules and 3D descriptors marked in the cubic grid**.

The grid points E_86, E_943 and S_482 had a positive contribution (38.318%, 28.499% and 9.023% respectively) towards the activity of thiosemicarbazones against cathepsin L, while the descriptor E_463 contributed negatively. Steric descriptors are related to both the size and shape of the molecules and fragments and all the bulk descriptors can be regarded as steric descriptors. A positively contributing steric descriptor signifies the importance of the presence of a bulky group at that position. As can be seen in the grid box (Figure 4), S_482 owing to its proximity to the bulky benzophenone moiety in the cubic grid suggests its importance at that site as activity enhancer. Electrostatic descriptors describe the importance of the presence of electronegative and electropositive groups at a site. Positively contributing electrostatic descriptors signify the importance of electropositive groups and negatively contributing ones signify the importance of electronegative groups. E_86 and E_943, both having positive contribution, lie relatively far away from the electronic cloud of the molecule. The presence of electronegative groups at R1 benzophenone site is therefore a necessity given the electropositivity enhancing descriptors lying far away. The third electrostatic descriptor E_463 contributes negatively and therefore acknowledges the presence of a highly electronegative group like halogens, O or N at the R1 benzophenone site for activity enhancement. Thus the R_1 _aromatic ring must have electronegative groups attached in order to increase the activity, for which compounds A1 and A19 are good examples having a highly electronegative fluorine atom attached at the 2^nd ^position. Compounds A7 and A18 with bulkier electronegative substituent at the 3^rd ^position are few other examples.

### Pharmacophore model

Pharmacophore development from a given set of molecules with high inhibitory activity against a particular protein target is a highly viable approach in ligand based drug design. It is done by using fine-grained conformational sampling and an array of scoring techniques to identify highly potent therapeutics. A pharmacophore conveys minimal characteristics of the structures of the ligands which are critical for binding to the target. Each hypothesis is accompanied with a set of aligned conformations that suggest the mode in which molecules are likely to bind relatively [48]. After selecting the maximum number of sites to be 5 (each site is involved in a 2-3 Kcal/mol interaction with the receptor), alignments were generated using model ligands that are a part of the active set of the series. The compounds A30, A35 and B14 (refer Additional file 1) were not selected out of total 29 (Additional file 1) for alignment thus reducing the number of matches to 26 for all the hypotheses. The hypotheses obtained along with their survival scores and selectivity are reported in Table 3.

**Table 3:** Statistical values of all the pharmacophore hypotheses generated for virtual screening

Row	ID	Survival	Survival-inactive	Selectivity	Matches
1	ADDRR.4	3.691	1.131	1.526	26
2	ADDRR.8	3.693	1.130	1.548	26
3	ADDHR.6	2.588	0.843	1.649	26
4	ADDHR.12	2.997	0.843	1.668	26
5	ADDHR.18	2.956	0.819	1.687	26
6	ADDHR.15	2.809	0.737	1.731	26
7	ADDHR.16	2.748	0.747	1.737	26
8	ADDHR.20	2.708	0.706	1.745	26
9	ADHRR.4	2.812	0.780	1.781	26
10	ADHRR.6	2.796	0.755	1.837	26
11	ADHRR.10	2.781	0.743	1.865	26
**12**	**DDHRR.8**	**2.808**	**0.756**	**2.058**	**26**
13	DDHRR.12	2.792	0.741	2.068	26

### Scoring function

In order to assign a score, each pharmacophore along with its ligand are temporarily regarded as the reference and other non-reference pharmacophores are aligned one by one using a least square procedure. Further a site score, a vector score and a volume score are calculated with combined weights for each aligned pharmacophore. Pharmacophoric hypotheses were scored on the basis of how good the alignment exists between the active set molecules and pharmacophoric features. After choosing the hypothesis for each box, the final scoring is done and the resultant is called the survival score of a hypothesis that characterizes its validity and potential to be used for a given set of molecules. The survival score constitutes a number of various scores and weights calculated during hypothesis generation as presented in equation 2.

(2) $$S=W_{site}S_{site}+W_{vec}S_{vec}+W_{vol}S_{vol}+W_{sel}S_{sel}+W_{rev}^{m}-W_{E}\;\Delta E+W_{act}\;A$$

where W's are the weights and S's are the scores

We selected a common pharmacophore hypothesis comprising of common chemical features of the aligned active molecules from the congeneric set. The final hypothesis, DDHRR.8 was chosen based on high selectivity as well as the survival score which yields the best alignment of the active set ligands. Along with the site score (0.318008), vector score (0.908012) and volume score (0.581835) DDHRR.8 was the best choice for searching a compound library.

**Figure 5:**
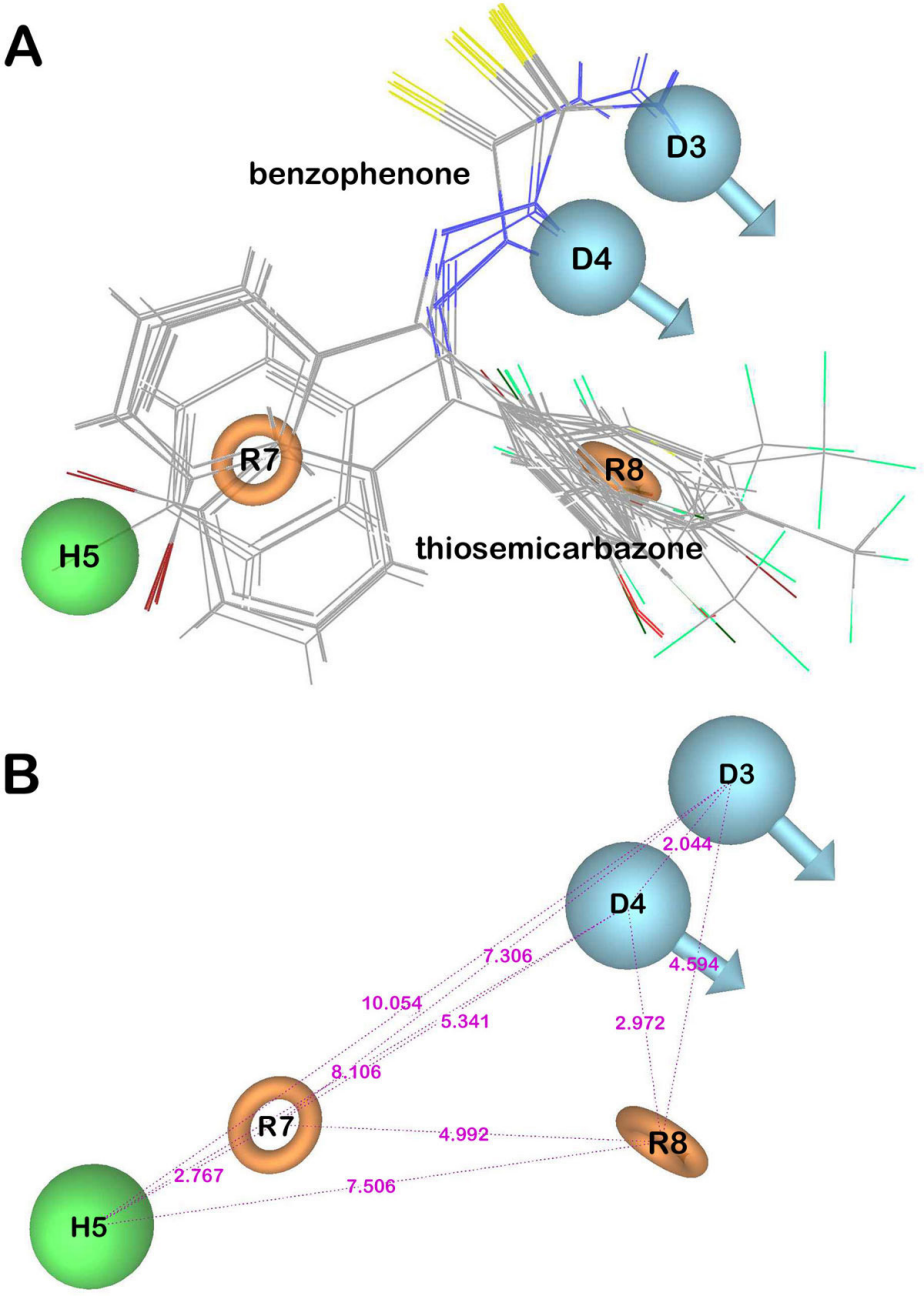
**Alignment of dataset molecules along with pharmacophoric features of DDHRR.8**.

Clearly, the name DDHRR implies the presence of two hydrogen donors, one hydrophobic group and two aromatic rings. In Figure 5 the hydrogen donors are marked with light blue spheres centered on the H atom with the arrows directing towards potential H-bonds and the aromatic rings marked as group sites represented by orange torus (ring), located at the centroid of a group of atoms. These marked sites give an idea about the mode of interaction of a lead molecule with Cathepsin L. As represented in Figure 5(a), an alignment of the 26 compounds from the congeneric series with that of the chosen hypothesis, DDHRR.8, supported its selection as the common pharmacophore hypothesis. In Figure 5(b), intersite distances (distances between pharmacophore site pairs) have been shown. Similar hypotheses were grouped together according to their intersite distances to identify the common pharmacophore.

### Pharmacophore based screening to identify anti-cancer leads

On screening the compound library based on the pharamacophoric hypothesis DDHRR.8, the resulting 7409 compounds were subjected to XP docking against cathepsin L (PDB ID 2YJ2) http://www.rcsb.org. The resultant 6 compounds that scored above the threshold were selected. The 3D-QSAR model developed using the same congeneric series as that of the pharamacophoric model was used to predict the activity of the resultant 6 compounds. The docking scores and predicted activities are summarised in Table 4. We report the top two scoring compounds obtained and evaluated through this combined approach.

**Table 4:** Top scoring compounds screened using the selected pharmacophore hypothesis

Compound ID	XP score	Align Score	Vector Score	Volume Score	Fitness	Predicted activity (using 3D QSAR model)
ZINC08764437	-7.972908	1.195091	0.567087	0.348039	0.919217	5.729
ZINC03846634	-7.575686	0.974276	0.229897	0.310700	0.728700	5.750
ZINC03846477	-7.222795	0.996626	0.614281	0.438287	1.222046	5.701
ZINC35415799	-7.173709	0.887849	0.369075	0.334232	0.963432	5.760
ZINC13570446	-7.071575	0.961697	0.405166	0.327059	0.930810	5.7553
ZINC13570446 (conformer)	-7.054554	0.628301	0.303334	0.325527	1.105277	5.790

**Figure 6:**
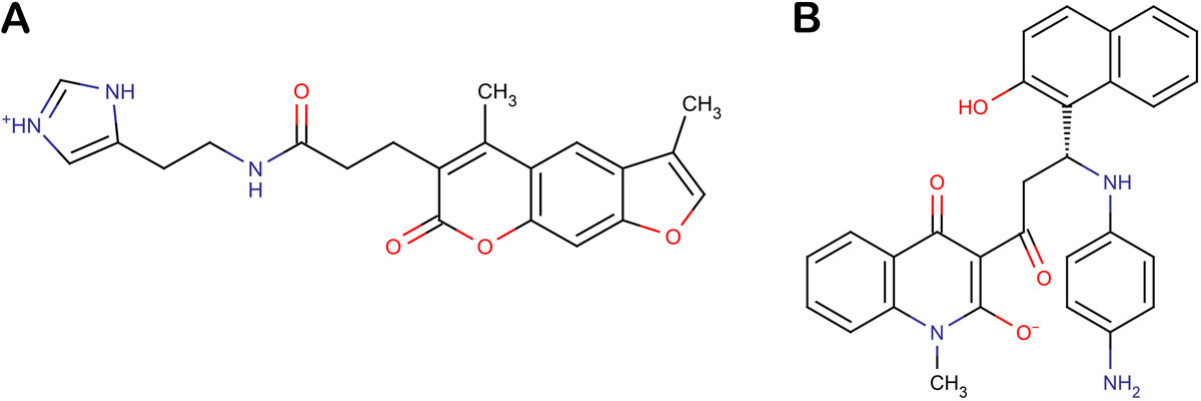
**Chemical structures of screened natural molecules (a) NFP (b) APQ**.

**Figure 7:**
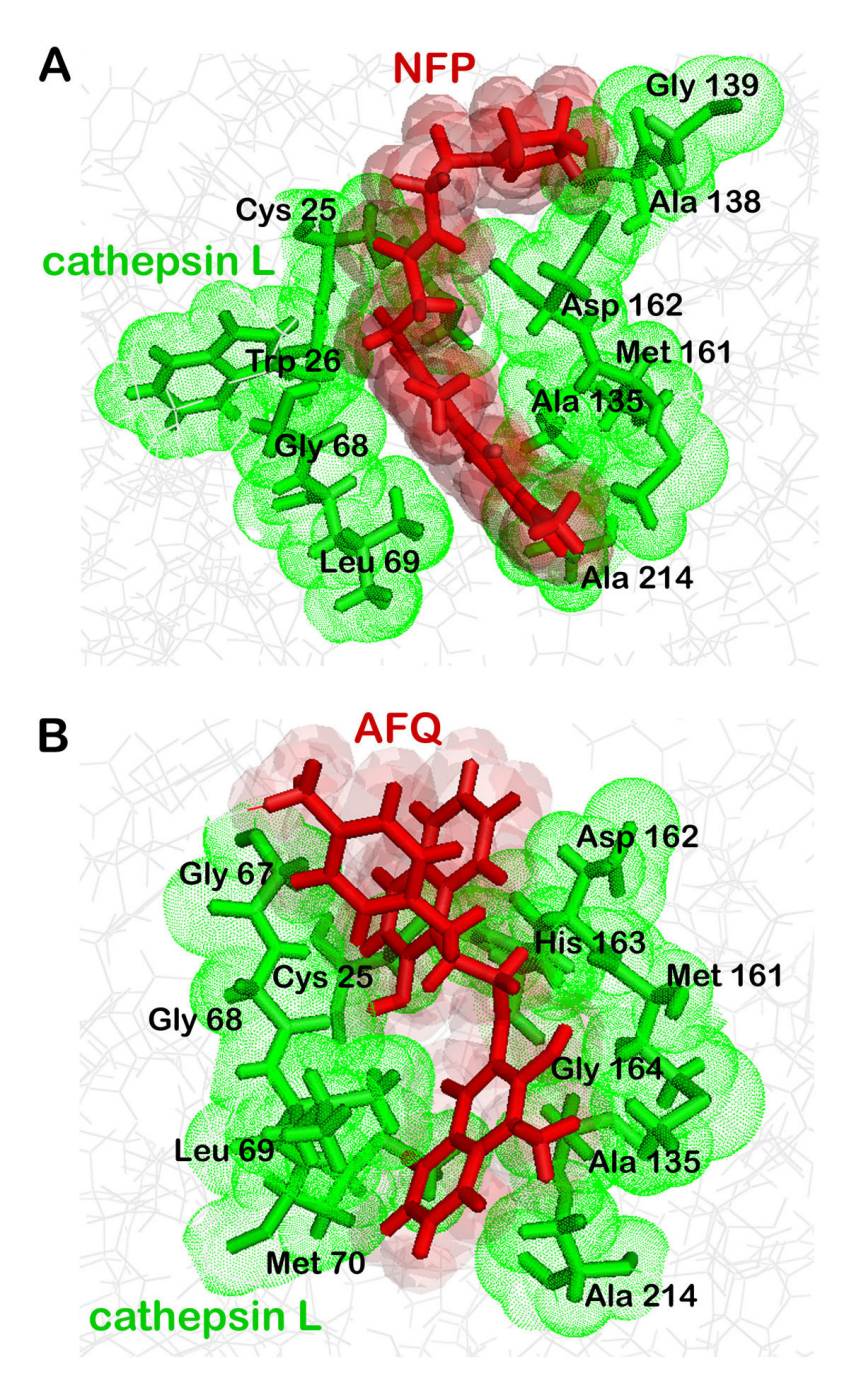
**Hydrophobic interactions between cathepsin L and screened compounds (a) NFP (b) APQ**.

The XP score gives the extent of binding affinity of the respective lead molecules with Cathepsin L, all of them lying under the specified threshold. We focused on the catalytic triad comprising of residues Cys25, Met161 and Asp162 and analyzed the interactions taking place between Cathepsin L and the thiosemicarbazone series (Figure 6). The first compound reported [*N-(2-(1H-imidazol-4-yl) ethyl)-3-(3,5-dimethyl-7-oxo-7H-furol[3,2-g] chromen-6-yl)propanamide*] (NFP, ZINC08764437) is a bulky ringed structure that interacted with the catalytic triad along with other residues: Ala138, Gly139, Trp26, Gly68, Ala135, Gly164, Leu69, and Ala214 (Figure 7A). The next top scoring candidate [*3-[3-(4-aminophenyl)amino-3-(2-hydroxy-1-naphthyl)-propanoyl]-4-hydroxy-1-methyl-quinolin-2-one*] (APQ, ZINC03846634), again a bulkier one, weakly interacted with the catalytic triad apart from Gly67, Gly68, Leu69, Met70, His163, Ala135 and Ala214 (Figure 7B). It can be inferred that due to the steric hindrance caused by its bulky aromatic groups, APQ fails to interact closely with Cys25, Met161 and Asp162. The align score refers to the extent of similarity with the chosen hypothesis, DDHRR.8. Align score was found to be highest for NFP being 1.195091 while for APQ it was 0.974276. We predicted the activities of the top scoring compounds using the generated 3D-QSAR model. The high predicted activities of NFP (5.72) and APQ (5.75) suggested that it is worth to consider them potent cathepsin L inhibitors.

## Conclusion

We used a combined approach to screen potent cathepsin L inhibitors that promised to emerge as important leads in cancer research owing to the role that cathepsin L plays during tumor development and metastasis. A congeneric set belonging to the thiosemicarbazone class of molecules which are known to inhibit human cathepsin L was chosen to build a 3D-QSAR model and a pharmacophore model. The former related the structure of the molecule with its activity quantitatively while validating the relationship using statistical parameters whereas the later pointed out the minimal structural features critical for a molecule for its biological activity and also provided an insight into the mode of binding with the target. Using these two approaches of ligand based drug designing we screened a chemical library based on the pharamacophoric hypothesis and then predicted their activity using the 3D QSAR model. The compounds obtained after pharmacophore-based search were docked at the active site (catalytic triad) of cathepsin L to further substantiate its role as a cathepsin L inhibitor. The two top scoring compounds NFP and APQ show good binding affinity with cathepsin L. This study presents a comprehensive view of the correlation between the structure and activity of these molecules along with their mode of binding with the target protein. This study progresses the use of thiosemicarbazone moiety as anti-tumoral and suggests further investigation into the role of human cathepsin L in the propagation of metastasis. Results of this study will also guide the design of potent anti-tumorals based on cathepsin L inhibition to further strengthen already available drug batch against cancer.

## Competing interests

The authors declare that they have no competing interests.

## Authors' contributions

CT, SoG and AG designed the methods and experimental setup. CT, SoG and JKD carried out the implementation of the various methods and were assisted by SG and MG. CT, SoG, JKD and AG wrote the manuscript. All authors have read and approved the final manuscript.

## Supplementary Material

Additional file 1 **This file includes the following table**. Table S1 - Structures and anti-cancer activities of thiosemicarbazone derivatives used in this study. Click here for file

## Declaration

AG would like to acknowledge financial support from Department of Science and Technology, Government of India towards publication of this article.

This article has been published as part of *BMC Genomics *Volume 14 Supplement 8, 2013: Selected articles from the International Conference on Intelligent Biology and Medicine (ICIBM 2013): Genomics. The full contents of the supplement are available online at http://www.biomedcentral.com/bmcgenomics/supplements/14/S8.
