# Supplementary material for: Mechanistic insights into mode of action of novel natural cathepsin L inhibitors
Source: BMC Genomics. 2013 Dec 9;14(Suppl 8):S10. doi: 10.1186/1471-2164-14-S8-S10 (PMC4042235; doi:10.1186/1471-2164-14-S8-S10)
Supplement: Additional file 1 — This file includes the following table. Table S1 - Structures and anti-cancer activities of thiosemicarbazone derivatives used in this study. [file 1471-2164-14-S8-S10-S1.PDF]

Additional file

Table S1 - Structures and anti-cancer activities of thiosemicarbazone derivatives used in this study

S.No.	Name	Structure	Cathepsin L IC_{50} (nm)	pIC_{50}	Predicted activity
1	A1	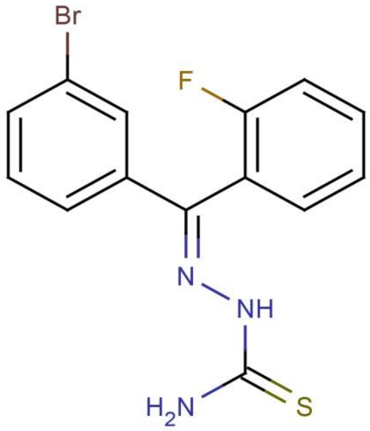	30.5	7.515	7.34785
2	A2	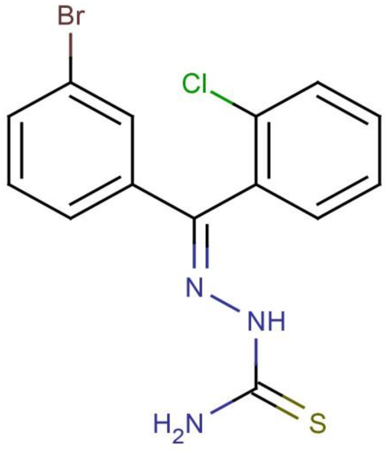	1610	5.793	5.73899
3	A6	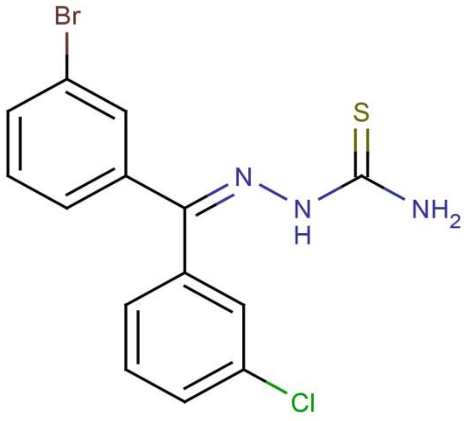	131	6.882	6.40231

4	A7	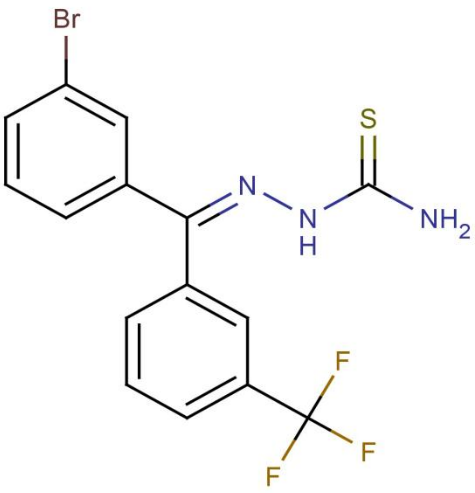	46.5	7.332	7.21309
5	A8	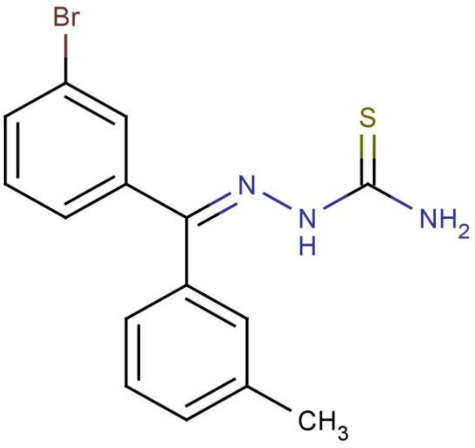	224	7.649	7.09503
6	A10	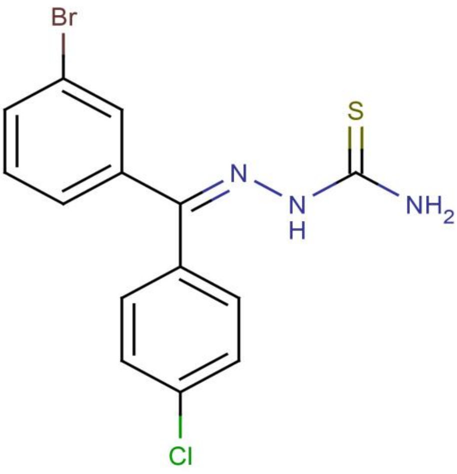	327	6.485	6.50621

7	A12	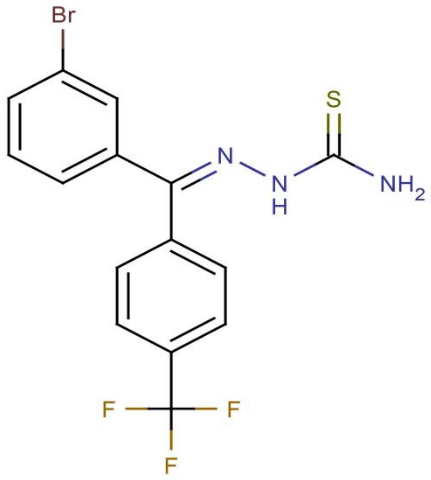	521	6.283	6.44789
8	A13	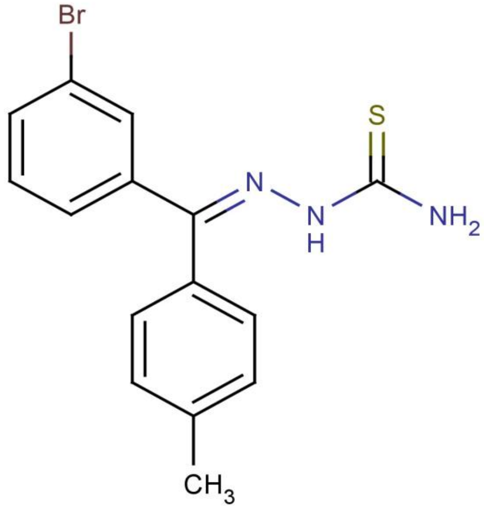	2160	5.665	5.75618
9	A14*	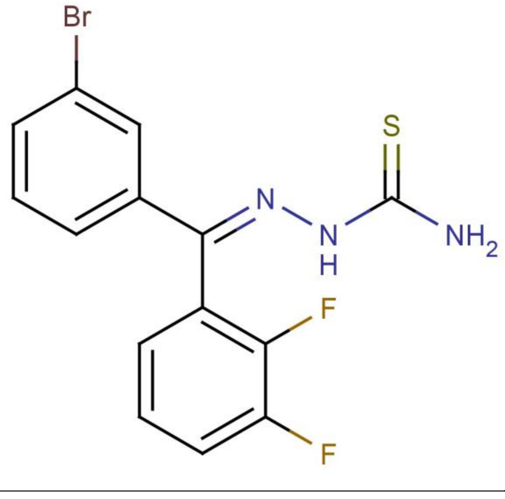	83.8	7.076	---------

10	A15	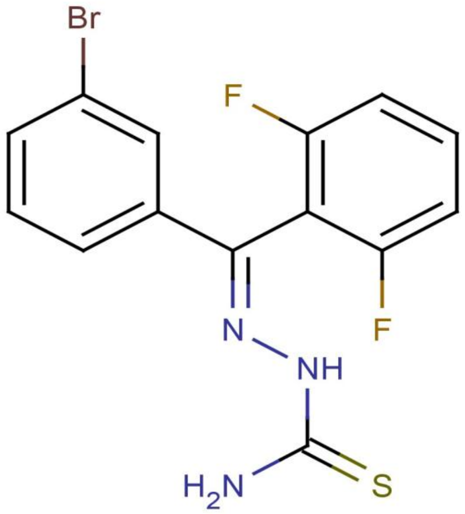	610	6.214	6.2753
11	A16	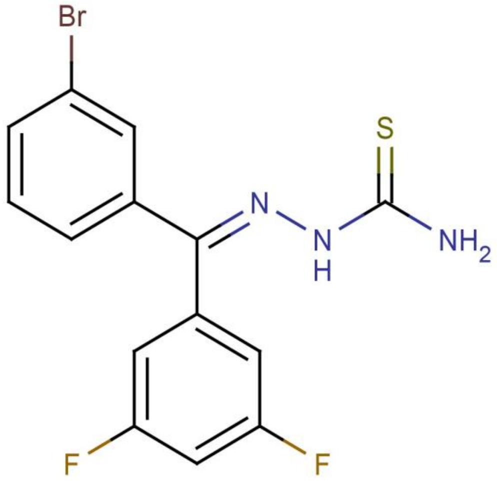	59.4	7.226	6.8636
12	A17	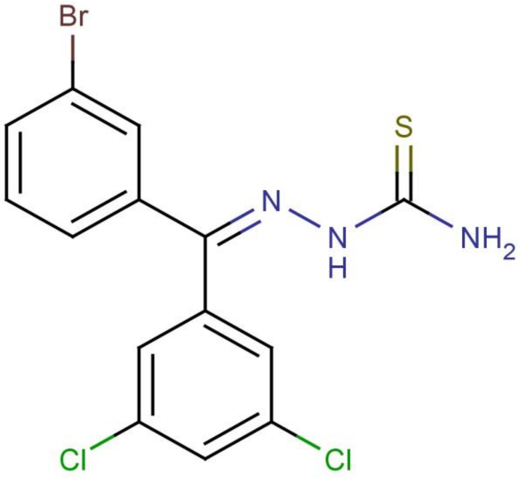	415	6.381	7.06119

13	A18	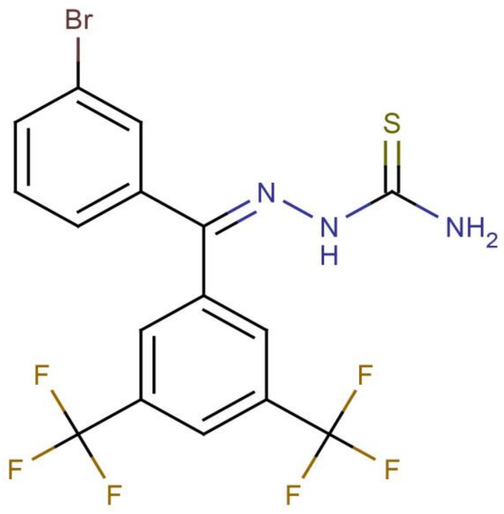	96.0	7.017	7.13568
14	A20	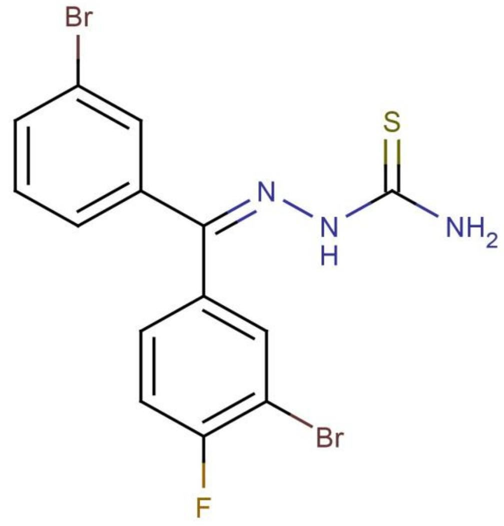	114	6.943	6.8764
15	A21	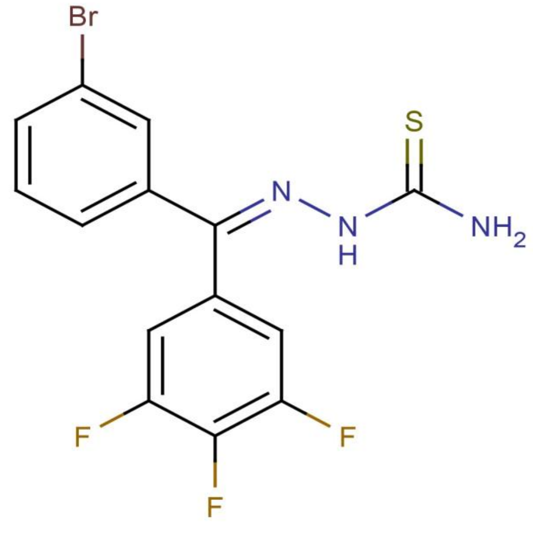	118	6.982	6.9029

16	A22	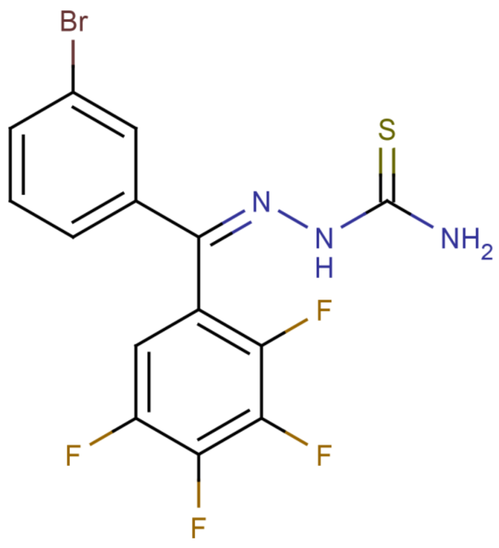	63.2	7.199	6.98515
17	A24	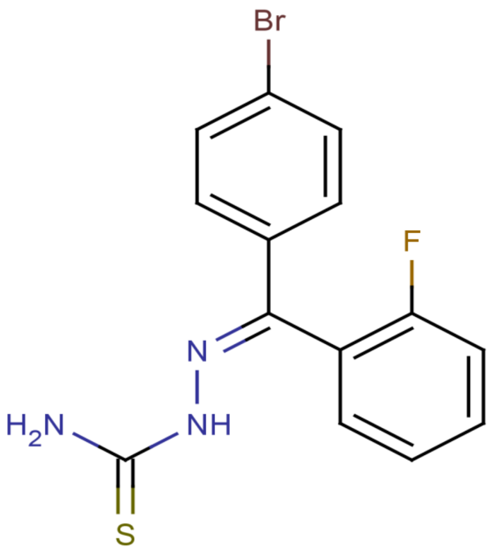	2220	5.653	5.62656
18	A26	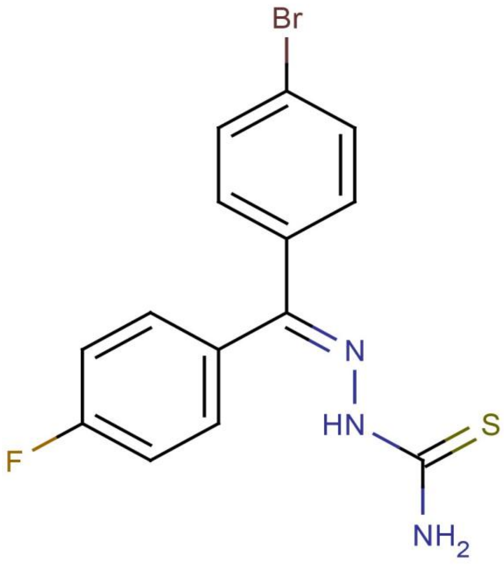	3320	5.478	5.48257

19	A30**	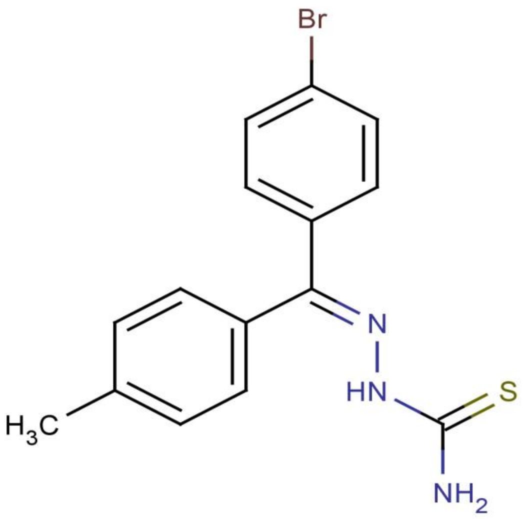	4570	5.340	5.10111
20	A35**	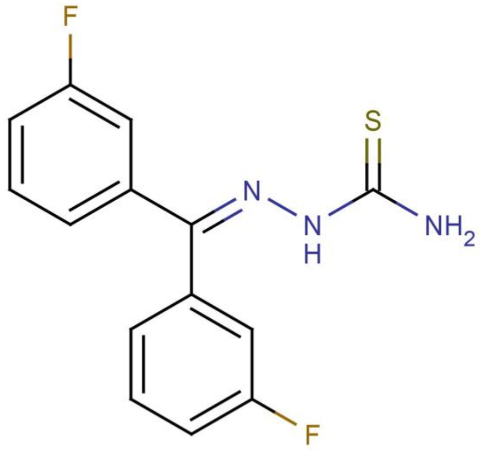	4870	5.312	5.86873
21	B2	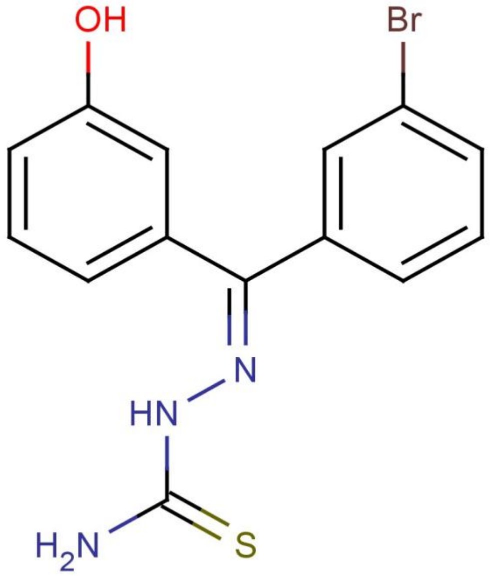	131.4	6.881	7.3739

22	B6	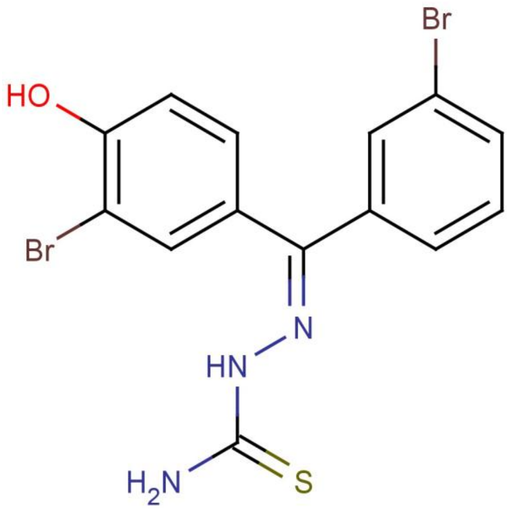	126.1	6.899	6.7173
23	B8	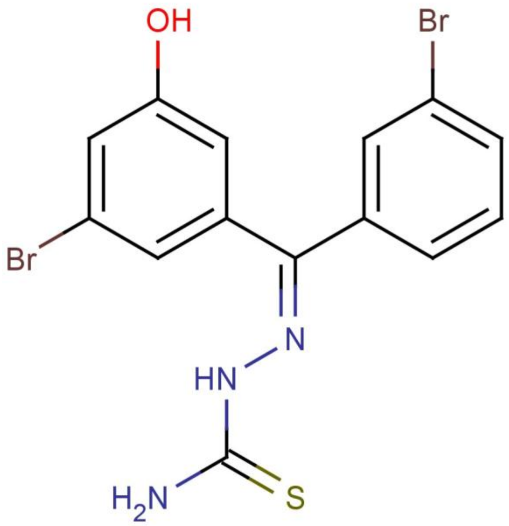	232.4	6.633	6.71506
24	B14**	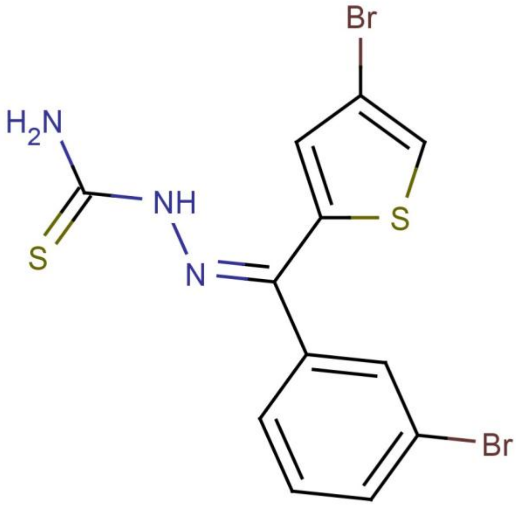		6.089	6.23662

25	A3	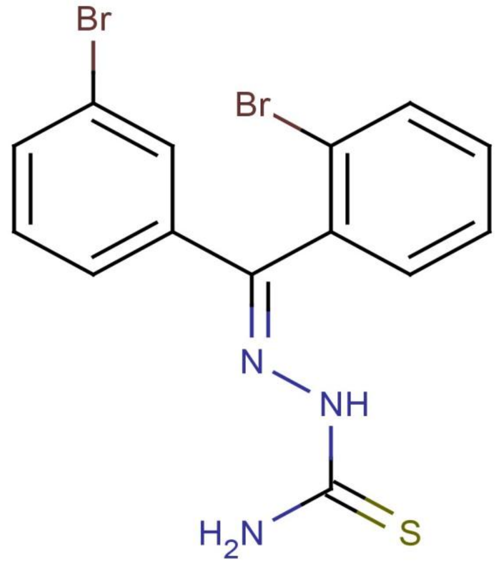	2600	5.585	5.14978
26	A5	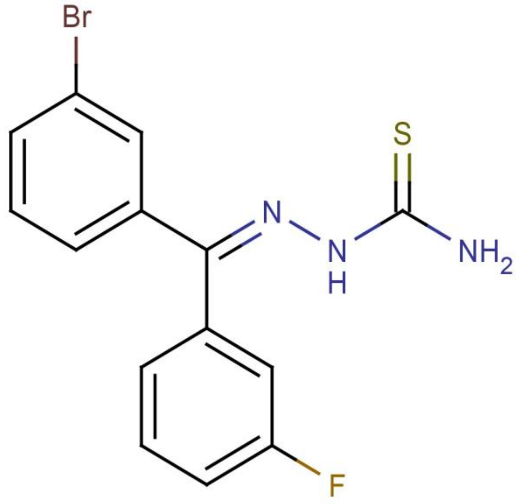	250	6.602	7.03431
27	A9	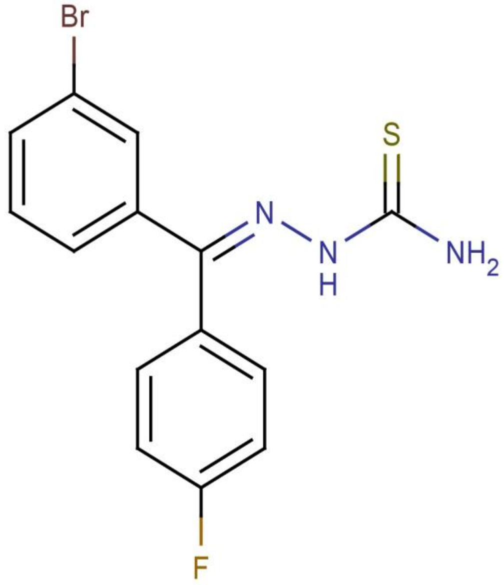	327	7.099	7.00561

28	A19	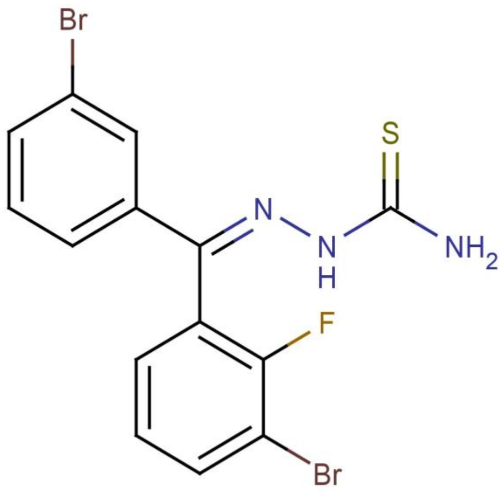	118	6.632	6.41696
29	A34	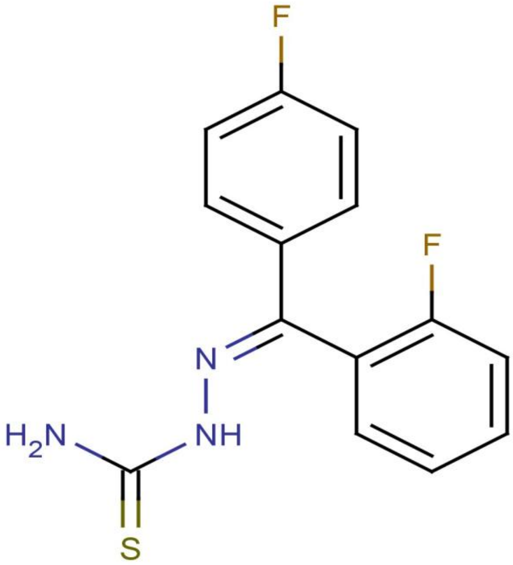	2460	5.609	5.29126

1. *Not used in 3D QSAR model development.
2. **Not used in pharmacophore model development.
